# Coping-Skills Training and Cue-Exposure Therapy in the Treatment of Alcoholism

**Published:** 1999

**Authors:** Peter M. Monti, Damaris J. Rohsenow

**Affiliations:** Peter M. Monti, Ph.D., is a professor of psychiatry and human behavior and associate director of the Center for Alcohol and Addiction Studies, Brown University, and a senior career research scientist at the Veterans Affairs Medical Center, Providence, Rhode Island. Damaris J. Rohsenow, Ph.D., is a research professor of community health and research director of the addictive behavior research laboratory at the Center for Alcohol and Addiction Studies, Brown University, and a career research scientist at the Veterans Affairs Medical Center, Providence, Rhode Island

**Keywords:** Coping skills, alcohol cue, AODU (alcohol and other drug use) treatment method, relapse prevention, patient education, treatment outcome, skills building, interpersonal skills, cognitive therapy, behavior therapy, AOD (alcohol and other drug) craving, social learning theory, literature review

## Abstract

Coping-skills training (CST) and cue-exposure treatment (CET) are two relatively new approaches in alcoholism treatment. With CST, the therapist tries to strengthen the patient’s skills in coping with situations associated with a high risk of drinking. These skills can be specific to certain high-risk situations or involve general social skills. Specific CST treatment approaches include relapse prevention training, social or communication skills training, urge-specific coping-skills training, and cognitive-behavioral mood management training. Several studies have shown that CST can be more effective than comparison treatments in improving the outcome (e.g., the frequency and severity of relapses) of alcoholic patients. CET exposes the patient to alcohol-related cues (e.g., the sight or smell of alcohol), thereby allowing the patient to practice responses to such cues in real-life situations. In addition, CET teaches a variety of coping skills for dealing with urges caused by such cues. Few studies have examined the effectiveness of CET, but the existing results demonstrate favorable treatment outcomes (e.g., reduced drinking severity).

In recent years, several exciting advances have provided clinicians with new tools in alcoholism treatment that have resulted in improved outcomes (e.g., lower relapse rates, reduced drinking levels, and improved health status) for alcoholic patients. Two of these new tools are coping-skills training (CST) and cue-exposure treatment (CET). CST aims to enhance the patient’s coping skills and provide him or her with specific strategies for coping with the urge to drink. Conversely, CET exposes the patient to alcohol-related cues (e.g., alcoholic beverages) during therapy, thereby allowing the patient the opportunity to practice using coping skills in response to the urge to drink within the safe environment of a treatment setting. Researchers hypothesize that as a result of coping-skills practice, patients will feel less overwhelmed by urge-provoking situations and, therefore, less likely to relapse after treatment. This article reviews the conceptual bases and methods of CST and CET and also summarizes the results of outcome studies that have assessed the effectiveness of both approaches.

## Coping-Skills Training

### Conceptual Overview

CST and the related treatment approach of social skills training have evolved from several decades of research based on social learning theory. According to social learning theory, several factors can increase the likelihood that an alcoholic will relapse when confronted with a stressful situation or with another situation that is associated with a high risk of drinking (e.g., attending a party where alcohol is served). Influential factors include limited skills in coping with stressful or high-risk situations, expectations that alcohol will have a positive or pleasurable effect in these situations (i.e., positive outcome expectancies), and the belief that the person cannot effectively cope with the situation without drinking (i.e., low self-efficacy expectations) (Marlatt and Gordon 1985).

Skills training is designed to address the aforementioned risk factors in several ways. First, clinicians can train patients in using coping skills specific to certain high-risk situations (e.g., refusing drink offers) to improve the patients’ skillfulness in handling similar situations in the future. Second, therapists can teach their patients general social skills that will result in improved sober relationships and reduced conflict in both family and work relationships. This improvement in social skills can diminish both the drinkers’ stress levels and the number of high-risk situations they encounter while simultaneously increasing their social supports for abstinence. Third, as a result of stronger coping and social skills, patients will likely develop increased self-efficacy expectations and, consequently, be more likely to effectively utilize those skills in high-risk situations.

Several lines of evidence support the importance of skills training for alcoholics. First, studies indicate that alcoholics’ coping skills are inferior to the coping skills of nonalcoholics, particularly in situations that commonly pose a risk of relapse, such as a family conflict or parties at which alcohol is served (Monti et al. 1989). Second, the skill levels that patients display in role plays of high-risk situations predict patient outcome after alcoholism treatment. For example, Monti and colleagues (1990) found that patients with low levels of coping skills in role plays consumed more alcohol during their first 6 months after treatment than did patients who had developed strong coping skills. Similarly, low skill levels, as measured by an inventory of coping skills during treatment followup, also predicted relapse during the subsequent 2 months (Miller et al. 1996). Third, alcoholics with low self-efficacy or with a high urge to drink during role plays of high-risk situations drank more during the 6 months following treatment (Monti et al. 1990) than did their high self-efficacy, low-urge counterparts.

Social learning theory also suggests that several types of personal and environmental factors and situations place a drinker at a particularly high risk for relapse. An understanding of these high-risk situations can help treatment providers design skills training programs that teach their patients the specific skills needed to cope with those situations.

One personal factor that contributes to a high risk of relapse is the lack of ability to control one’s positive (e.g., excitement and euphoria) and negative (e.g., depression and anger) emotional states (Monti et al. 1995). Analyses of high-risk situations for relapse have shown that negative emotional states in particular precede many relapse episodes (Marlatt and Gordon 1985). Consequently, enhanced social and coping skills could prevent relapse in interpersonal high-risk situations (e.g., conflict with others) as well as ameliorate relapse risk in intrapersonal high-risk situations (e.g., boredom, loneliness, and depression). Chronic life stress (e.g., from a death in the family or a stressful job) can exacerbate many of these high-risk situations (Monti et al. 1995).

Another common risk factor for relapse is the presence of alcohol or drinking-associated stimuli (i.e., alcohol cues), which may be combined with either direct or indirect peer pressure (Marlatt and Gordon 1985; Monti et al. 1995; Niaura et al. 1988). Consequently, the drinker must acquire the skills necessary to avoid or “walk away from” such high-risk situations and develop a healthy lifestyle (e.g., sober friendships and recreational activities not involving alcohol) that will decrease the probability of exposure to alcohol cues. A drinker cannot avoid all alcohol-related cues, however. For example, he or she may see other people drink in public or social settings. Such situations can disrupt the drinker’s ability to use his or her coping skills effectively. Consequently, CST must take into account alcohol cues; for example, by incorporating CET, which is discussed in more detail in the second half of this article.

### CST Treatment Methods

All CST approaches generally begin with an assessment of the patient’s areas of vulnerability (Monti et al. 1995). This assessment can focus on several domains, such as coexisting biological or psychiatric conditions, intrapersonal and interpersonal risk factors, and the expected amount of exposure the patient will have to alcohol cues. Several biological or psychiatric conditions exist that if co-occurring with alcoholism can increase a patient’s risk for relapse or interfere with his or her effective use of coping skills. For example, in people with an affective disorder,^1^ emotional situations can be associated with a higher risk of relapse; accordingly, those patients require an enhanced focus on the management of their emotional states. Similarly, psychotic states or certain neurological conditions can impede coping by causing cognitive impairment.

Because some intrapersonal factors can influence relapse risk, the clinician should assess those that are relevant, including the patient’s current skill in controlling mood states, such as euphoria, anger, depression, and stress. In addition, the treatment provider should evaluate the extent to which situations involving various mood states have posed a risk of heavy drinking to the patient in the past.

At the beginning of CST, the clinician also should assess the patient for interpersonal risk factors, including general social skills, conflict resolution skills, and refusal skills, as well as the types of interpersonal situations that have contributed to heavy drinking in the past. Finally, the treatment provider must determine the extent to which the patient will be exposed to alcohol cues. For example, patients whose jobs involve being around alcohol (e.g., waiters) or whose family or extended social network includes drinkers or alcoholics may require cue-specific coping skills or CET.

A valuable way to obtain specific information about these various risk factors, particularly for outpatients, who are not treated in a controlled environment, is to have the patients themselves monitor both high- and low-risk situations by maintaining a detailed daily log. Such logs can help the treatment provider and the patient identify both the risk factors and the protective factors to which the patient is exposed. Although clinicians have developed several types of CST, the following four approaches have been used most extensively (for a more detailed description, see Monti et al. 1995):

- *Relapse prevention training*, in which each treatment session focuses on a specific type of situation that poses a high risk for relapse and teaches various skills to use in that situation
- *Social or communication skills training*, in which each treatment session focuses on a general interpersonal skill designed to improve social relationships; this approach strives to reduce conflicts, improve the alcoholic’s sober supports, and change his or her lifestyle
- *Urge-specific coping-skills training*, which is described later within the context of CET
- *Cognitive-behavioral mood management training*, in which each treatment session focuses on skills for managing specific emotions and which is discussed in more detail in the article in this issue by Longabaugh and Morgenstern, pp. 78–85.

The following sections describe the first two approaches.

#### Relapse Prevention Training

This CST approach, originally developed by Chaney and colleagues (1978), typically includes eight sessions, each of which generally focuses on only one type of situation that poses high risk for relapse. These high-risk situations fall into four major categories: (1) frustration and anger (e.g., interpersonal conflict), (2) interpersonal temptation (e.g., an offer of a drink), (3) negative emotional states (e.g., depression, boredom, and loneliness), and (4) intra-personal temptation (e.g., craving or finding a bottle of an alcoholic beverage). The various high-risk situations, as well as the appropriate coping skills for dealing with them, have been described in detail by Marlatt and Gordon (1985).

Relapse prevention training can be conducted in either group or individual sessions. In either setting, each session includes a variety of components, as follows:

- Direct instruction in effective coping skills for specific situations
- Modeling of those skills by therapists and/or group members
- Rehearsal of the behaviors in role plays
- Feedback about the patients’ responses and asking the patients about their thoughts
- Instructions in the cognitive process used in generating the responses (e.g., thinking through possible alternative responses and the consequences of each response).

Through this process, the patients are taught to define the situational problem by identifying its relevant elements (e.g., wanting to retain the friendship of a person offering a drink but also wanting to avoid drinking), to generate several possible responses, and to think about the consequences of each response. To practice their new skills, participants discuss and role play a sample situation before identifying specific personal examples of that type of situation. Each patient role plays his or her specific high-risk situation and receives feedback on how to enhance the effectiveness of his or her response. The patient then repeats the same role play using the new and more effective response. In addition, the patient reports what he or she was thinking when generating the response so that thoughts or emotions such as anger or hopelessness can be addressed.

#### Communication Skills Training

In contrast to relapse prevention training, communication skills training does not focus on high-risk situations but, instead, focuses on communication skills that can be used to handle a variety of risky situations (Monti et al. 1990). This approach addresses at least eight high-priority skills that are discussed in separate training sessions. These skills include (1) refusing a drink, (2) giving positive feedback, (3) giving criticism effectively, (4) receiving criticism about alcohol and other drug use, (5) developing listening skills, (6) improving conversation skills, (7) developing sober supports, and (8) learning effective approaches to conflict resolution. Five additional skills that can be covered in separate sessions, if time permits, include (1) nonverbal aspects of communication, (2) expression of feelings, (3) introduction to assertiveness, (4) request refusal, and (5) management of criticism in general. However, these skills also can be covered within the first eight modules (for more information, see Monti et al. 1989).

Communication skills training generally is conducted in a group setting. Each session begins with a summary of the meeting’s goals and the reason why the skill is important for sobriety, followed by a brief presentation and discussion of the skill involved. Depending on the participants’ initial skill level, the therapist can role play a sample vignette illustrating the skill to be discussed. The patients then generate personal examples of possible scenarios in which the skill may be needed. As with relapse prevention training, the sessions include instruction in effective coping skills, modeling of those skills by therapists and group members, behavior rehearsal in role plays with feedback, and coaching of responses and of cognitive aspects of the response.

### Evidence for the Effectiveness of CST

Several studies have evaluated CST’s effectiveness in improving patient outcome and have compared it with the effectiveness of other approaches. Miller and colleagues (1995), in a comprehensive review of various treatment methodologies, found that several studies supported the effectiveness of social coping-skills training. For example, one study among alcoholics receiving inpatient treatment in a Veterans Affairs (VA) hospital compared the effectiveness of three approaches—relapse prevention training, a discussion group, and no additional treatment—all of which were delivered in addition to standard inpatient therapy (Chaney et al. 1978). In that study, patients who had received relapse prevention training experienced relapses of significantly lower severity and duration than did patients in the two control groups.

A second study (also conducted among alcoholics receiving inpatient treatment in a VA hospital) compared communication skills training, either for the alcoholic alone or for both the alcoholic and his or her family members, with another approach, cognitive-behavioral mood management training (Monti et al. 1990). Again, the communication skills training, regardless of whether it involved family members, resulted in less frequent and less intense drinking than did the alternative treatment approach. The investigators in that study also conducted patient-treatment matching analyses to determine which type of therapy might be most effective for which type of patient. Those analyses found that patients who had achieved a comparatively higher education level or who had experienced relatively low urges and less anxiety benefited equally from communication skills training and mood management training (Rohsenow et al. 1991). Conversely, patients who had achieved a comparatively low education or who had experienced relatively high urges or anxiety only benefited from communication skills training. These findings suggest that compared with cognitive-behavioral mood management training, skills training may be useful for a broader range of patients.

Measures of patients’ skill levels as determined by standardized tests (e.g., process measures) gathered during some of these studies provide further information about the mechanisms underlying CST’s beneficial effects. For example, in the study by Chaney and colleagues (1978), patients receiving CST generated longer and more specific responses in standardized role plays of high-risk situations (e.g., meeting an old friend who suggests going to a bar) than did patients in the control groups. Furthermore, the time it took a patient to make any response in these role plays strongly predicted the patient’s outcome (i.e., duration and frequency of relapse). These observations suggest that one of the most important results of treatment is that in future high-risk situations, the patient is able to rapidly generate a coping response.

Monti and colleagues (1990) used similar measures to determine the mechanisms underlying treatment effectiveness. In that study, alcoholics who received communication skills training showed greater improvement in the effectiveness of their responses to standardized role-play tests, particularly in the use of interpersonal strategies, than did patients receiving mood management therapy. Moreover, patients who exhibited more effective skills and quicker responses in role plays at the end of treatment drank less over the next 6 months than did patients without these characteristics. Thus, these studies indicate both that CST results in improved coping skills and that a patient’s ability to respond quickly and effectively in role plays of high-risk situations can predict reduced drinking severity after treatment.

Other studies, such as the Project MATCH trial, however, did not find greater benefits of skills training approaches compared with other approaches. In the multisite Project MATCH study, alcoholic patients who either had completed inpatient therapy (i.e., the aftercare sample) or had received only outpatient care (i.e., the outpatient sample) were randomly assigned to receive either cognitive-behavioral therapy (i.e., skills training), motivational enhancement therapy, or 12-step facilitation. This study did not find any differences in overall outcome between the three treatment approaches among either the aftercare or the outpatient sample (Project MATCH Research Group 1997). However, several factors limit the conclusions that can be drawn from these results:

- The cognitive-behavioral approach in the Project MATCH study combined communication skills training modules with cognitive-behavioral mood management training. As described previously, however, mood management training benefits only a limited number of patients and may thereby reduce the effectiveness of the overall cognitive-behavioral approach.
- The three treatment approaches in Project MATCH were administered only to outpatients (i.e., patients who had less severe alcohol problems) or to patients who had already successfully completed an intensive inpatient treatment program. Consequently, the study allows no conclusions regarding the effectiveness of these approaches when they are administered as a component of (i.e., concurrently with) intensive inpatient or partial hospitalization programs. Studies in which skills training was administered during such intensive programs generated more favorable results (Miller et al. 1995).
- Each of the treatment approaches in Project MATCH was the sole form of treatment that the patients received during the study period. In regular treatment settings, however, the various treatment approaches are commonly administered together with other measures as part of a full treatment program. Thus, skills training along with the other two approaches might be more beneficial if they were administered as one component in a comprehensive treatment program.
- No process measures assessing the acquisition of skills were collected during the Project MATCH study period; consequently, researchers cannot determine whether the patients actually learned the skills that they were taught. Without such measures, it is difficult to determine whether patient outcomes had not improved because the patients had learned new skills but those skills did not affect outcome or because the patients did not attain the necessary skills (i.e., the therapists were ineffective at teaching those skills). Other studies that did include process measures of skill acquisition reported beneficial results from skills training (e.g., Chaney et al. 1978; Monti et al. 1990).
- In Project MATCH, the skills training was conducted with individual patients. Researchers generally consider group therapy superior, however, because it allows for multiple sources of feedback (Monti et al. 1989). Consistent with that hypothesis, studies that conducted skills training in a group setting showed beneficial results from that approach (e.g., Chaney et al. 1978; Monti et al. 1990).

## Cue-Exposure Treatment

### Conceptual Overview

An alcoholic can encounter numerous alcohol-related cues in his or her environment. These cues can include the sight and smell of a favorite alcoholic beverage; mood states or situations in which drinking previously occurred; and people, places, times, and objects that had previously been associated with alcohol’s pleasurable effects. Two types of models—learning theory models and social learning theory—have been used to explain the relationship between alcohol-related cues and relapse to drinking.

Classical learning theory models of alcohol cues and relapse suggest that environmental cues that were associated with drinking in the past can elicit conditioned responses,^2^ which in turn may play a role in precipitating relapse (Niaura et al. 1988; Rohsenow et al. 1995). The exact nature of these responses, however, is still controversial. According to one hypothesis, they could be equivalent to conditioned withdrawal—that is, exposure to these cues would make the body “expect” alcohol and induce withdrawal-like symptoms when the expected alcohol is withheld. Alternatively, the cue-induced responses could be equivalent to conditioned compensatory responses (i.e., physiological responses that counteract alcohol’s effects) or conditioned appetitive responses (i.e., responses similar to those produced by alcohol itself). Current evidence suggests that cue-induced responses most resemble conditioned appetitive responses (Niaura et al. 1988).

In addition to classical learning theory models, social learning theory suggests that the presence of cues may increase the risk of relapse by increasing the relevance to the drinker (i.e., salience) of the positive effects of alcohol, which can make the drinker want to consume more alcohol. According to this theory, alcohol cues also can trigger cognitive and neurochemical reactions that may undermine a drinker’s ability to use coping skills as well as the drinker’s beliefs that he or she can effectively use those skills in the situation (Monti et al. 1995; Niaura et al. 1988). The explanations of both social learning and other learning theory models complement each other.

Several lines of evidence support the hypothesis that exposure to alcohol cues can increase the risk for relapse. For example, Monti and colleagues (1995) evaluated the reactions of alcoholics in response to holding and smelling a glass of their favorite alcoholic beverage as well as their performance in a drink-refusal role play that either did or did not involve their favorite beverage as a prop. In that study, drinkers who responded more strongly to their favorite beverage also performed more poorly if the role play included their favorite beverage as a prop than if the same role play did not include any props. This observation indicates that alcohol cues can disrupt a drinker’s ability to use coping skills, suggesting that such cues may increase the risk of relapse even in drinkers who are otherwise adept at using those skills. Other studies in which alcoholics were exposed to alcohol cues in a laboratory setting found that those cues impeded the alcoholics’ ability to pay attention to other stimuli (e.g., a certain sound), as indicated by increased reaction times in response to those stimuli (Sayette et al. 1994). These findings suggest that the presence of alcohol cues may reduce the ability of alcoholics to utilize their coping skills. Finally, Rohsenow and colleagues (1994) found that alcoholics who reacted more strongly (i.e., exhibited more salivation and less attention to the alcohol stimuli) when exposed to alcohol cues in a laboratory setting also drank more during followup.

These studies suggest several strategies for designing more effective treatment approaches. For example, treatment could be designed to reduce the degree of the reactions alcoholics experience in the presence of cues by repeatedly exposing the patients to those cues in a safe environment. As a result of such an approach, the disruptive effects of alcohol cues on attentional processes and on the ability to use coping skills should be reduced. Alternatively, treatments could provide alcoholics with practice in using coping skills while in the presence of alcohol cues. Such an approach should allow an alcoholic to use his or her coping skills more effectively even when experiencing a strong reaction to alcohol cues.

CET may exert its beneficial effects through two different mechanisms. First, learning theory posits that repeated presentation of a cue (e.g., the sight of a favorite drink) while preventing the usual response (e.g., drinking) should result in decreasing reactions across sessions (i.e., habituation) and possibly the permanent loss (i.e., extinction) of the elicited response (e.g., salivation or urge) over time. However, such habituated reactions are specific to the particular cues used. Accordingly, the reaction is easily reinstated if the alcoholic is exposed to a different cue (e.g., seeing his or her favorite bar). Second, social learning theory suggests that engaging in coping skills practice in the presence of alcohol-use cues should increase both the effectiveness of those skills when in the presence of cues and the drinker’s beliefs about his or her ability to respond skillfully when confronted by similar cues in real-life situations. As a result of this repeated practice, internal reactions to alcohol cues likely will interfere less with the drinker’s ability to use his or her coping skills in the future.

### CET Methods

Various approaches to CET exist (for a review, see Rohsenow et al. 1995). Historically, CET has been based on classical learning models, focusing on the habituation or extinction of responses (Niaura et al. 1988; Rohsenow et al. 1995). According to these models, the patient only needs to be exposed repeatedly to alcohol without being allowed to drink (i.e., undergo a series of nonreinforced exposures) in order to prevent the previous response (i.e., drinking) to those cues. Social learning theory suggests, however, that the chance to practice coping skills in the presence of alcohol cues also may be an important aspect of CET (Rohsenow et al. 1995). Consequently, some CET approaches have focused primarily on pure exposure to alcohol cues, whereas other approaches have included coping-skills training in the presence of alcohol cues.

Another variation among different approaches has been the nature of the cues that have been used during exposure trials (Rohsenow et al. 1995). For example, the settings associated with drinking are highly varied and may be idiosyncratic for individual drinkers. Accordingly, it is difficult to expose drinkers participating in studies or treatment to all possible real-life settings associated with drinking. To approximate the diversity of environmental and emotional cues involved, some CET approaches use imaginary exposure—that is, the patient is asked to imagine a situation in which drinking has occurred previously. This approach is particularly useful for cues or settings that cannot be reproduced in treatment sessions, such as fights or the death of a loved one.

Because the sight and smell of alcohol, in addition to being a powerful cue in its own right, is a cue that every drinker is exposed to before he or she drinks, many CET approaches let the patient hold and smell an alcoholic beverage. Some CET approaches—particularly those that are based on the idea that the response to be prevented is heavy drinking and that moderate drinking is acceptable—even go a step further and use the ingestion of a small amount of an alcoholic beverage as the cue (Rohsenow et al. 1995). Thus, the choice of alcohol cues to be used is based, at least in part, on treatment philosophy (e.g., on whether the treatment goal is abstinence or moderate drinking) and on the constraints of the setting in which CET is to be conducted (i.e., the extent to which real-life drinking settings can be recreated).

Various CET approaches have involved only cue exposure and were designed with a goal of moderate drinking (for a detailed review, see Rohsenow et al. 1995). Several of these studies involved alcohol administration to alcoholic patients. All the studies were conducted in Great Britain, where this approach is considered acceptable, although it is not acceptable in the United States. Specific treatment strategies used in these studies included the following:

- The patients sniffed, then tasted, and then drank 1-ounce drinks of bourbon while decreasing the size of sips and increasing the time between sips (Pickens et al. 1973).
- The patients consumed either one or four double vodkas, depending on the treatment day (Hodgson and Rankin 1976).
- The patients only observed and sniffed the beverage in some sessions and in other sessions were encouraged to drink no more than one “double” in different settings (e.g., the hospital, the patient’s home while watching television, or a pub) (Rankin 1982).
- The patients, who were experiencing alcohol-related problems but were not alcohol dependent, consumed three standard drinks^3^ for men and two standard drinks for women during each session, then looked at an additional drink while being instructed not to drink it. Some of the sessions were conducted in a room at the treatment facility, other sessions were conducted (as homework) in the patient’s home (Sitharthan et al. 1997).

Another approach, which was abstinence oriented, involved exposure to cues with no drinking allowed (Drummond and Glautier 1994). In 40-minute sessions conducted over 10 days, the patients were asked to “act out drinking” by picking up, looking at, and smelling a drink and thinking about drinking it. Based on the theory that habituation alone can be beneficial, this approach involved no coping-skills training.

Several treatment approaches have combined CET with urge-specific CST. In one such approach, which was aimed at achieving moderate drinking patterns, alcoholics received two drinks as a priming dose, then either looked at a third drink while being instructed to resist drinking or imagined being exposed to alcohol and successfully refusing the drink (Rankin et al. 1983). However, this approach involved only a limited use of coping skills and only during imagined alcohol exposure, but not when looking at a real drink.

A more elaborate approach, combining the learning and practicing of urge-specific coping skills with exposure to actual alcohol cues and to imagined high-risk situations, has since been developed for alcoholics in abstinence-oriented treatment programs (Monti et al. 1993; for a detailed description of the procedures, see Monti et al. 1995; Rohsenow et al. 1995). This treatment generally involves six or eight individual or group sessions and can be conducted in both inpatient and outpatient settings. The supervision of the treatment setting ensures that no actual drinking occurs. Furthermore, outpatients should stay in the treatment setting for several hours afterwards to ensure that any lasting reactions are safely handled by therapists. Before treatment initiation, the therapist conducts two types of assessment:

- *Cue reactivity assessment*, in which the patient holds and smells a glass of his or her usual alcoholic beverage while salivation, urge to drink, and other responses are measured
- *Drinking Triggers Interview*, a questionnaire used to identify the situations in which the patient experiences the strongest urges to drink.

Each treatment session begins with the alcoholic holding a glass of his or her usual alcoholic beverage; smelling it, if that will increase the urge; and focusing on the aspects of the beverage that produce the strongest urge. During that time, the patient reports every change in the level of urge in terms of a 0 to 10 scale. Once the patient’s urge level has peaked (usually after 1 to 5 minutes), one of two events occurs. In the first session, the patient is asked to continue focusing on the beverage to “see what happens to your urge.” Experience has shown that the urge generally decreases within 15 minutes, an effect that usually is quite different from what the patient had expected. In all later sessions, the patient is asked to imagine using the urge-specific coping strategy that he or she was taught most recently to reduce the urge.

After the part of each session that involves actual beverage exposure, one or more trials involving imaginary exposure are conducted in the same session, using similar procedures. In these trials, the patient is asked to imagine being in one of the high-risk situations identified in the Drinking Triggers Interview, starting with the situation that had received the highest urge rating. After the patient’s urge has peaked, he or she is asked to continue to imagine being in the situation and to either “wait out” the urge (first session only) or apply the coping skill most recently taught. A variety of different urge-specific coping skills are taught in the sessions, including the following:

- Using delay as an active cognitive coping tool—that is, the drinker tells himself or herself that the urge will “go away” if he or she just “waits it out”
- Thinking about the negative consequences of consuming alcohol in the imagined situation
- Thinking about the positive consequences of staying sober in the imagined situation
- Using urge-reduction imagery developed by Marlatt and Gordon (1985), such as imagining slashing the urge with a sword
- Using behavioral substitution—that is, imagining engaging in an alternative activity
- Using consummatory substitution, such as consuming favorite foods and sodas instead of alcohol
- Employing mastery statements, such as “I am strong; I can get through this without drinking”
- Using pleasant imagery—that is, imagining escaping to a pleasant place.

This CET approach incorporates both the classical learning theory and social learning theory aspects. Thus, an alcohol cue (i.e., the alcoholic beverage) is present throughout the treatment session, both during times when the alcoholic is actively focusing on the cue (i.e., active exposure) and during all other parts of the session (i.e., passive exposure). Habituation or extinction processes come into play as a result of exposure without drinking. In addition, alcoholics are taught useful coping skills and can practice using those skills while their urges are elevated, either by alcohol exposure or by imagining being in high-risk situations.

Social learning theory predicts several results of this approach:

- Practicing coping skills while experiencing an urge for alcohol should decrease the likelihood that future urges will disrupt the use of coping skills in the real-life environment.
- Alcoholics will experience the benefits of coping skills because their urges will decrease more rapidly when they apply the skills. This experience should increase both the patients’ confidence that they can apply the skills and their expectancies that the urge will decrease if they use the coping skills.
- This procedure should allow treatment providers to tailor the coping skills to be used in high-risk situations to the individual needs of each patient. Thus, patients are asked to try various skills for coping with each drinking trigger situation during treatment and to identify the skills that are most effective for each particular trigger situation. At the end of treatment, each patient then receives a printed copy of his or her individualized “tool box” for future reference.

### Evidence of the Effectiveness of CET

Few controlled CET studies—that is, studies in which researchers compared CET’s effectiveness with that of other treatment approaches—have been conducted. Two studies assessed CET’s effectiveness when drinking was allowed during treatment because moderate drinking was a treatment goal. Rankin and colleagues (1983) compared the effectiveness of actual alcohol cue exposure with that of imaginary alcohol exposure. The investigators first provided a small group of patients with two drinks, then either exposed the patients to an additional drink (i.e., had them look at a drink) or asked them to imagine that they were exposed to, and successfully refused, an additional drink. In the study, actual alcohol exposure to alcohol cues was more effective than imaginary exposure and refusal in terms of decreasing the alcoholics’ urge to drink and increasing their self-efficacy. However, the investigators did not collect any outcome data (e.g., drinking levels after treatment).

More recently, Sitharthan and colleagues (1997) compared the effectiveness of a CET approach aimed at moderate drinking with the effectiveness of cognitive-behavioral training focused on developing moderate drinking patterns in drinkers who were not alcohol dependent. Moderate drinking in this study was defined as two drinks for women or three drinks for men per occasion. Thus, female patients in the CET group were allowed to consume two drinks and male patients were allowed to consume three drinks before being exposed to an additional drink. Patients who had received the CET approach showed greater reductions in drinking quantity and frequency than did patients who had received cognitive-behavioral treatment.

Two additional controlled studies of CET were conducted in abstinence-oriented programs and therefore did not involve any alcohol consumption. In one study, CET (i.e., exposure to alcohol cues) was compared with a control treatment involving relaxation training, both provided in addition to standard inpatient treatment (Drummond and Glautier 1994). In that study, alcohol-dependent patients who had received CET exhibited less drinking severity during followup than did patients who had received relaxation training.

Monti and colleagues (1993) compared the effectiveness of two therapy approaches: (1) CET combined with urge-specific CST, which was provided in addition to standard inpatient treatment, and (2) standard inpatient treatment only. All patients also received two assessment sessions that included passive exposure to alcohol cues. The researchers followed the patients for 6 months. During the first 3 months of followup, both patient groups performed equally well in terms of drinking frequency and severity. During the second 3-month followup period, however, patients who had received CET were more likely to remain completely abstinent, experienced more abstinent days, and tended to have fewer drinks when they drank compared with control patients. Thus, two controlled studies have demonstrated that CET—both with and without the inclusion of urge-specific CST—can produce favorable treatment outcomes in alcoholics in terms of reducing drinking severity.

Measures of the variables the treatments were intended to affect (i.e., process measures) gathered during the study conducted by Monti and colleagues (1993) have shed some light on the mechanisms that might account for the aforementioned results. For example, the researchers found that alcoholics in both the CET group and the control group showed decreased responses to alcohol cues (i.e., decreased urges to drink) during treatment, although this decrease was greater in the CET group than in the control group. Because urge levels at the start of treatment by chance had been higher in the CET group than in the control group, however, the researchers do not know whether differences in treatment or differences in patient characteristics accounted for the differences in urge reduction and treatment outcome. Another factor that influenced treatment outcome was the subjects’ self-efficacy. Thus, patients with more confidence in their ability to cope with high-risk situations at discharge drank less during followup than did patients with less confidence. However, confidence levels increased similarly in both the CET and the control groups.

Monti and colleagues (1993) also found that patients who had received CET reported more frequent use of some urge-specific coping skills during followup than did patients who had received the control therapy. Moreover, the frequency with which these skills were used—particularly those of thinking about the negative consequences of drinking and the positive consequences of sobriety—correlated with decreases in drinking. These observations suggest that the beneficial effects of the CET approach may have resulted in part from the urge-specific skills training component and provide guidance about which skills might be most useful. Furthermore, the findings of this study underscore that the collection of process measures during controlled studies is important for guiding the development of future treatment interventions and for refining existing ones.

## Conclusions

As the studies reviewed in this article indicate, CST has a strong track record of improving drinking outcomes when used in combination with comprehensive alcoholism treatment programs. Thus, CST approaches, regardless of whether they focus on coping with specific high-risk situations or on general communication skills, have resulted in improved skills and decreased drinking severity. Few controlled trials have evaluated the effectiveness of CET, however, although the studies that do exist indicate that CET is a promising approach and should be investigated further. Moreover, those studies have demonstrated that although cue exposure alone can result in favorable outcomes, the opportunity to practice urge-specific coping skills while experiencing cue-induced urges may be particularly beneficial. Researchers are currently developing and investigating additional approaches for combining skills training with cue exposure, and the results of those investigations should provide guidance for clinicians in the future.

Researchers also are investigating the usefulness of therapy approaches that combine CST and CET with pharmacotherapy involving the medication naltrexone. This medication is one of only two agents approved for alcoholism treatment and has been shown to reduce relapse rates in alcoholics who are also receiving psychosocial therapy. Naltrexone acts on brain chemicals involved in mediating alcohol’s pleasurable effects and may thereby reduce a drinker’s desire to consume alcohol. Preliminary results of a recently completed study have suggested that naltrexone treatment can significantly reduce the number of patients reporting any urge to drink during alcohol exposure (Monti et al. in press). These findings suggest that cue reactivity methodology (e.g., studying patient’s responses to alcohol cues) may be useful in determining potential mediators of the therapeutic effects of pharmacotherapies as well as of behavioral treatments. Given the consistently favorable effects of CST and CET in reducing urges in alcohol-abusing and alcohol-dependent patients, a systematic examination of the effectiveness of combining these approaches with naltrexone therapy appears warranted.

In summary, the past decade has seen important advances in effective behavioral and, more recently, pharmacological treatment approaches for alcoholism. This progress will likely continue in the coming years, particularly as various promising approaches are combined to develop more effective ways of treating alcohol dependence.

## Footnotes

1 An affective disorder is any one of a group of psychiatric disorders that are characterized by severe and inappropriate emotional responses, prolonged or persistent mood disturbances, or other symptoms associated with depressed or manic states. Affective disorders include, for example, depression and bipolar disorder.

2 A conditioned response occurs when a stimulus that elicits a specific response is repeatedly paired with a neutral stimulus which normally does not elicit that response. If the neutral stimulus is paired frequently enough with the response-inducing stimulus, it becomes a conditioned stimulus that now also can elicit the response (which has now become a conditioned response). For example, in the classical experiment of Pavlov’s dog, food served as a stimulus that elicited a response of salivation. If the dog heard the sound of a bell (i.e., the neutral stimulus) every time the dog was exposed to food, the sound eventually became a conditioned stimulus that also could elicit a conditioned response of salivation even in the absence of food.

3 A standard drink is defined as 12 ounces of beer or wine cooler, 5 ounces of wine, or 1.5 ounces of distilled spirits.
